# The crystal-induced activation of NLRP3 inflammasomes in atherosclerosis

**DOI:** 10.1186/s41232-017-0050-9

**Published:** 2017-09-11

**Authors:** Tadayoshi Karasawa, Masafumi Takahashi

**Affiliations:** Division of Inflammation Research, Center for Molecular Medicine, Jichi Medical University, 3311-1 Yakushiji, Shimotsuke, Tochigi 329-0498 Japan

**Keywords:** Cholesterol, Cytokines, Inflammation, Interleukin-1, Macrophages

## Abstract

Atherosclerosis is an inflammatory disease, which is accompanied by the deposition of cholesterol-rich lipids and the infiltration of macrophages. Other well-known features of atherosclerotic lesions include the deposition of cholesterol crystals and calcium phosphate crystals; however, their pathophysiological role remains unclear. Recent studies suggest that cholesterol crystals play a pivotal role in activation of NLRP3 inflammasomes, which regulate caspase-1 activation and the subsequent processing of IL-1β, in atherosclerotic lesions. NLRP3 inflammasomes are essential for the initiation of vascular inflammation during the progression of atherosclerosis. Therefore, the regulatory mechanisms of NLRP3 inflammasomes are regarded as potential targets for atherosclerosis treatment. Here, we review the current knowledge regarding the role of NLRP3 inflammasomes in the progression of atherosclerosis and the prospects for therapeutic approaches targeting NLRP3 inflammasomes.

## Background

Atherosclerosis is an inflammatory disease characterized by the deposition of cholesterol-rich lipids and the macrophage infiltration of the vascular walls [1]. Infiltrated macrophages uptake cholesterol and cause inflammatory responses by producing various cytokines and chemokines. The mechanism of cholesterol accumulation in atherosclerotic lesions has been well described. For example, infiltrated macrophages incorporate modified low-density lipoprotein (LDL) via scavenger receptors and accumulate in the atherosclerotic lesions as lipid-loaded foam cells [2]. However, the molecular mechanisms by which lipids induce inflammatory responses are not been fully understood. In particular, the deposition of cholesterol crystals is a well-known feature of atherosclerosis [3], although the precise role of cholesterol crystals in the pathophysiology of atherosclerosis remains unclear. Recently, the molecular complexes called nucleotide-binding oligomerization domain-like receptor (NLR) family, pyrin domain containing 3 (NLRP3) inflammasomes have emerged as a key player for the crystal-induced inflammation in atherosclerosis [4, 5].

NLRP3 is a pattern recognition receptor (PRR), which participates in innate immune responses by recognizing danger signals, including pathogen-associated molecular patterns (PAMPs) and damage/danger-associated molecular patterns (DAMPs) [6]. The PRRs are evolutionarily conserved and expressed in the cells involved in the innate immune system such as macrophages, neutrophils, and dendritic cells [7]. The PRRs are classified into several groups according to their conserved structure and function. For instance, toll-like receptors (TLR) and C-type lectin receptors (CLRs) are expressed on the membrane surface, while NLRs and retinoic acid-inducible gene-I-like receptors (RLRs) exhibit intracellular localization. Recently, the involvement of several PRRs in the progression of atherosclerosis has been unveiled. Among PRRs, TLR2 and TLR4 are activated by inflammatory lipids, such as oxidized LDL and saturated fatty acids [1, 2]. Furthermore, NLRP3 is involved in the cholesterol crystal-mediated inflammatory response through the assembly of molecular complexes called inflammasomes. NLRP3 inflammasomes regulate inflammatory responses via processing of interleukin (IL)-1β, which is a potent inflammatory cytokine. Indeed, previous reports suggest that the development of atherosclerosis development was attenuated in the absence of NLRP3 inflammasomes [4, 8–10]. In this review, we describe the molecular mechanisms of crystal-mediated NLRP3 inflammasome activation and the regulatory mechanisms of NLRP3 inflammasomes in the atherosclerotic lesions.

## What is the inflammasome?

**Fig. 1:**
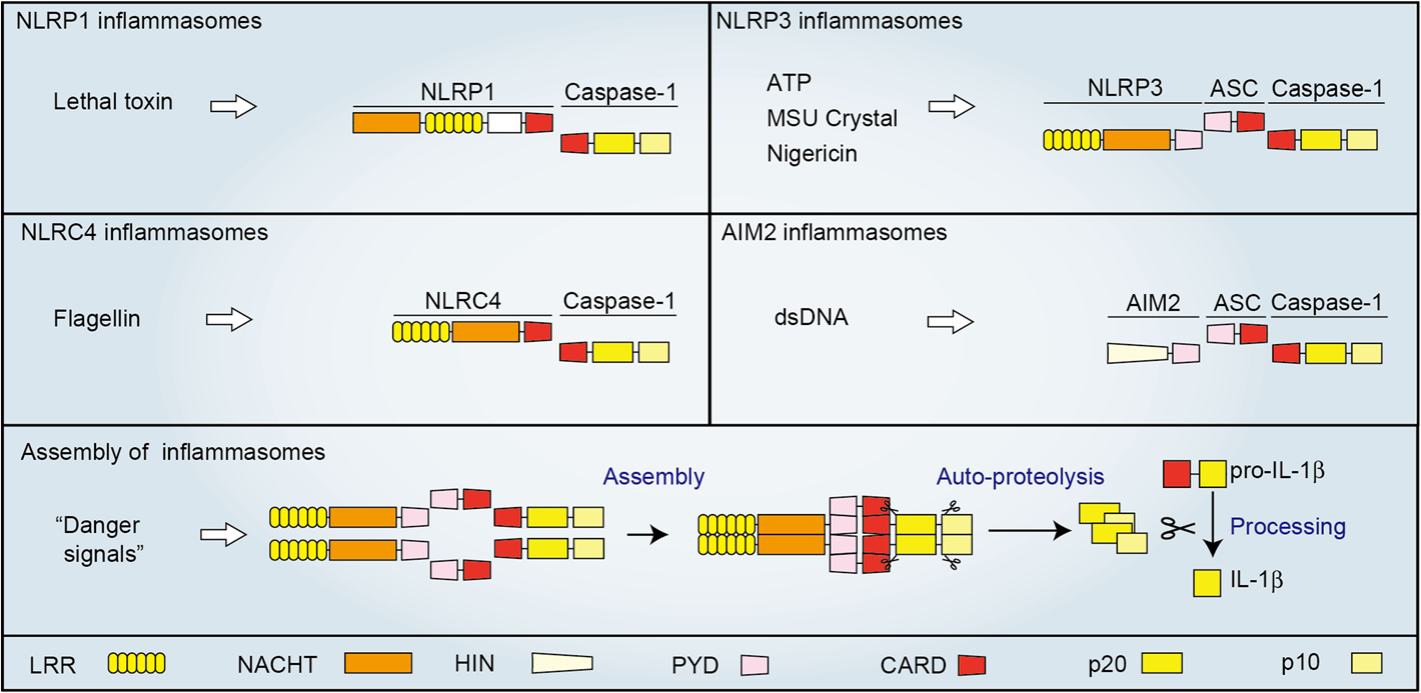
Components of inflammasomes. Several PRRs, which recognize distinct DAMPs, form the inflammasome complex that serves as a molecular platform for caspase-1 activation. NLRP1 inflammasomes are composed of NLRP1 and caspase-1 and are activated by lethal toxins. The components of NLRP3 inflammasomes are NLRP3, ASC, and caspase-1. NLRP3 binds ASC via PYD–PYD interaction. ASC subsequently binds caspase-1 via CARD–CARD interaction. NLRP3 inflammasomes are activated by both PAMPs and DAMPs, such as adenosine triphosphae (ATP), nigericin, and monosodium urate (MSU) crystals. NLRC4 inflammasomes are composed of NLRC4 and caspase-1 and are activated by flagellin. AIM2 inflammasomes are composed of AIM2, ASC, and caspase-1 and recognize double-strand DNA (dsDNA)

Inflammasomes are multiple cytoplasmic protein complexes, which are typically composed of NLRs, apoptosis-associated speck-like protein containing a caspase recruitment domain (CARD) (ASC), and caspase-1 (Fig. 1). In response to DAMPs or PAMPs, components of inflammasomes assemble through the interaction of pyrin domain (PYD) and caspase recruitment domain (CARD) [11]. Assembled inflammasomes form large molecular complexes and serve as a molecular platform for caspase-1 activation. Caspase-1 is a cysteine protease, which was originally identified as an interleukin-1 converting enzyme [12]. Therefore, inflammasome-mediated activation of caspase-1 converts inactive pro-IL-1β to its mature form. Among NLRs, at least NLRP1, NLRP3, NLRC4, NLRP6, and NLRP12 participate in inflammasomes as core components of the complexes [11]. Besides NLRs, PYHIN (pyrin and HIN domain-containing protein) family proteins, including absence in melanoma 2 (AIM2) and IFN-g-inducible protein 16 (IFI16), are also known as core components of inflammasomes. The adaptor protein ASC is necessary for several core components, such as NLRP3 and AIM2, for inflammasome assembly, while NLRC4 can assemble without ASC. Inflammasomes are named according to their core components. For example, the complexes composed of NLRP3, ASC, and caspase-1 are called NLRP3 inflammasomes. The core components of inflammasomes are activated by different danger signals. NLRC4 inflammasomes are activated by flagellin, a protein derived from flagellum of bacteria [13], while NLRP1 also recognizes, a lethal toxin derived from bacteria [14]. AIM2 functions as a sensor molecule for viral infection by recognizing cytosolic double-strand DNA [15]. NLRP3 is distinct from other core components because NLRP3 is activated by both DAMPs and PAMPs, while other core components are mainly activated by PAMPs and are involved in infection [6].

## NLRP3 inflammasomes

NLRP3 inflammasomes are activated by DAMPs as well as PAMPs and thus involved in both sterile inflammation and host defense [6]. DAMPs, including extracellular adenosine triphosphate (ATP) and monosodium urate (MSU) crystals, induce the assembly of NLRP3 inflammasomes to activate caspase-1 (Fig. 1). The interaction among components of NLRP3 inflammasomes is mediated by conserved domains, which exhibit homophilic interaction. NLRP3 contains three domains: C-terminal leucine-rich repeats (LRRs), a central nucleotide domain termed the NACHT domain, and an N-terminal PYD. ASC can function as an adaptor molecule because it is composed of an N-terminal PYD and a C-terminal CARD. Caspase-1 also has a CARD and catalytic domains (p10 and p20). When cells are exposed to danger signals, NLRP3 assembles homotypically by the NACHT domain and offers scaffold for filamentous assembly of ASC by their interaction of PYD [16]. Subsequently, the assembled ASC promotes recruitment of caspase-1 via CARD–CARD interaction and subsequent auto-activation of caspase-1.

The activated caspase-1 exerts proinflammatory effects by its proteolytic activity. Besides IL-1β, caspase-1 converts pro-IL-18 to its bioactive mature form. Furthermore, caspase-1 also cleaves gasdermin D (GSDMD) to induce pyroptosis, an inflammatory programed cell death accompanied by an increased permeability of the plasma membrane [17, 18]. Because IL-1β has no signal sequence for exocytosis, pyroptosis-mediated membrane permeabilization seems to be necessary for IL-1β release [19, 20].

**Fig. 2:**
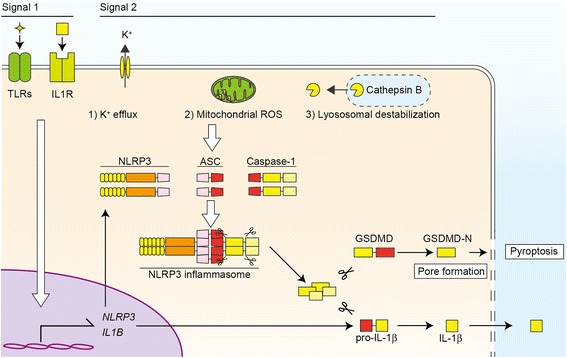
Mechanisms of NLRP3 inflammasome-driven IL-1β release. IL-1β release is regulated by a two-step regulation: transcriptional synthesis of pro-IL-1β and proteolytic processing into its mature form by the inflammasomes. The transcriptional regulation of IL-1β mRNA is mediated by TLRs and IL-1 receptor (signal 1), which also induces NLRP3 mRNA expression. Then, NLRP3 inflammasomes induce caspase-1 activation and the subsequent conversion of pro-IL-1β to its mature form (signal 2). As common upstream pathways of NLRP3 inflammasomes, three mechanisms are known: (1) potassium efflux, (2) generation of mitochondrial ROS, and (3) lysosomal destabilization and leakage of cathepsin B. Activated caspase-1 also cleaves GSDMD, whose processed N-terminal fragment (GSDMD-N) forms plasma membrane pores to increase membrane permeability, resulting in pyroptosis

The release of IL-1β is induced by a two-step regulation: transcriptional regulation called the priming step and processing by inflammasomes (Fig. 2). The transcriptional regulation of *Il1b* (signal 1) is mediated by PRRs or cytokine receptors including TLRs and IL-1 receptors (IL-1R). Besides NF-κB-mediated mRNA induction of *Il1b* and *Nlrp3*, activation of these receptors primes NLRP3 by posttranscriptional regulation, such as ubiquitination and deubiquitination [21, 22]. Then, accumulated cytosolic pro-IL-1β in cytosol is rapidly processed by caspase-1, which is activated by NLRP3 inflammasomes (signal 2). Since IL-1β exhibits potent proinflammatory effects, this two-step regulation is thought to be necessary for maintenance of immuno-homeostasis.

Although various endogenous or exogenous danger signals such as ATP, MSU crystals, and silica are known to activate NLRP3 inflammasomes, the precise mechanism by which NLRP3 recognizes the danger signals remains unclear [23, 24]. Unlike other PRRs, the direct ligands of NLRP3 are almost unknown and still controversial. Only a few reports suggest that mitochondrial DNA or mitochondria-derived cardiolipin functions as a direct ligand of NLRP3. Conversely, upstream molecular machineries of NLRP3 inflammasomes have been elucidated. Several common pathways including potassium (K^+^) efflux, generation of mitochondrial reactive oxygen species (ROS), and lysosomal destabilization are necessary for the activation of the NLRP3 inflammasome [6]. In particular, lysosomal destabilization and subsequent cathepsin B release is a common pathway for NLRP3 inflammasome activation by crystals and particulate matter. Thus, the regulatory mechanisms of NLRP3 inflammasomes have not been completely elucidated.

## NLRP3 inflammasomes in the pathogenesis of diseases

Originally, NLRP3 was identified as a responsible gene for cryopyrin-associated periodic syndrome (CAPS), which includes three syndromes with differing severity [25]. Familial cold autoinflammatory syndrome (FCAS) is the mildest condition and is characterized by cold-induced fever and inflammation. Muckle–Wells syndrome (MWS) is a moderate condition and is characterized by episodic attacks with fever and urticaria-like rash. MWS patients also exhibit arthralgia and progressive hearing loss. Chronic infantile neurological cutaneous and articular syndrome (CINCA) is the most severe condition with continuous inflammation, which results in neurological impairment. Since CAPS is a rare genetic disease, few investigations of NLRP3 inflammasomes had been performed until the link between NLRP3 inflammasomes and sterile inflammation was uncovered. However, the discovery that the NLRP3 inflammasome is activated by MSU crystals and associated with gout highlights the role of NLRP3 inflammasomes in sterile inflammatory diseases [23]. Indeed, we and others have revealed a pivotal role of NLRP3 inflammasomes in the development of cardiovascular and renal diseases including atherosclerosis [4, 8, 26–29]. Furthermore, in the last decade, numerous studies revealed that NLRP3 inflammasomes are activated by a broad variety of danger signals and are involved in various inflammatory diseases [6].

## Crystal-induced NLRP3 inflammasome activation

Among danger signals that activate NLRP3 inflammasomes, crystals and particulate matter share similar molecular mechanisms to activate NLRP3 inflammasomes. Innate immune cells, including macrophages and neutrophils, engulf these particles to remove foreign substances. However, excess loads of particles in lysosome cause an indigestion called “frustrated lysosome,” which in turn induces lysosome destabilization. As described above, the leakage of cathepsin B into the cytosol triggers NLRP3 inflammasome activation. Although downstream mechanisms of cathepsin B release are still unclear, K^+^ efflux is regarded as essential for the activation of NLRP3 inflammasomes induced by lysosomal destabilization [30, 31].

**Fig. 3:**
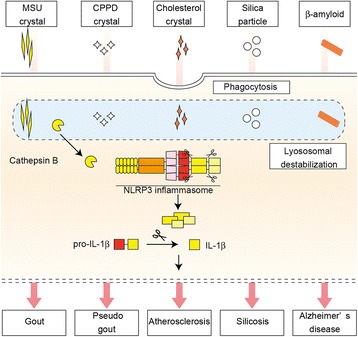
NLRP3 inflammasome activation mediated by lysosomal destabilization. Various endogenous crystals and exogenous particulate matter activate NLRP3 inflammasomes and cause inflammatory diseases. Phagocytosed particles cause lysosomal destabilization and rupture, which induce the leakage of lysosomal enzyme cathepsin B, resulting in the activation of NLRP3 inflammasomes. As a factor that induces lysosome destabilization, the following crystals or particles are known: MSU crystals, calcium pyrophosphate (CPPD) crystals, cholesterol crystals, silica particles, and β-amyloid

NLRP3 inflammasome activation induced by crystals and particles is associated with various inflammatory diseases (Fig. 3). As endogenous crystals, MSU crystals and calcium pyrophosphate dehydrate (CPPD) crystals are known to activate the NLRP3 inflammasome and cause inflammation in gout and pseudo-gout, respectively [23]. Exogenous particles such as silica and asbestos are associated with inflammation in silicosis and asbestos lung [32, 33]. However, factors that induce lysosomal destabilization are not limited to crystals and particulate matter. Some kinds of protein aggregates also activate NLRP3 inflammasomes. β-amyloid associated with Alzheimer’s disease is capable of activating NLRP3 inflammasomes [34]. Furthermore, NLRP3 inflammasome complex in the extracellular space can function as a danger signal of NLRP3 inflammasomes itself. The assembly of NLRP3 inflammasome components causes the formation of aggregates called speck, which are released to the extracellular space after NLRP3 inflammasome activation [35, 36]. The released NLRP3 inflammasomes are incorporated into neighbor cells in which they activate NLRP3 inflammasomes in part by lysosomal destabilization. Thus, various kinds of particles including crystals activate NLRP3 inflammasomes and initiate inflammatory responses.

## NLRP3 inflammasomes in atherosclerosis

In the advanced atherosclerotic lesion, the deposition of cholesterol crystals and calcium phosphate crystals are reported [3]. However, their pathophysiological relevance to atherosclerosis development remained unclear. In 2010, Douwell et al. [4] revealed that cholesterol crystals are deposited even in the early stage of atherosclerotic lesion and activate NLRP3 inflammasomes via lysosomal destabilization. Interestingly, they further showed that oxidized LDL, a major lipid species in atherosclerotic lesions, not only induces cholesterol crystallization but also provides priming signals to induce NLRP3 and pro-IL-1β expression. Thus, it is suggested that oxidized LDL could be sufficient to provide signals 1 and 2 to induce IL-1β release. According to a subsequent report by Sheedy et al. [37], incorporation of oxidized LDL via scavenger receptor CD36 provokes intracellular crystallization of cholesterol. On the other hand, calcium phosphate is also particulate matter, which is accumulated in atherosclerotic lesions and associated with vascular calcification. In this regard, we and other investigators showed that calcium phosphate crystals, including hydroxyl apatite and tricalcium phosphate, activate NLRP3 inflammasomes through lysosomal rupture and subsequent cathepsin B release [8].

Since several studies have shown that IL-1β contributes to the progression of atherosclerosis [38, 39], it is expected that the deficiency of NLRP3 inflammasomes prevents atherosclerosis. Indeed, recent studies reported that the deficiency of NLRP3 inflammasome components prevents the atherosclerosis progression [4, 8–10]. However, Menu et al. [40] reported that deficiency of NLRP3 inflammasomes failed to prevent the development of atherosclerosis in ApoE^−/−^ mice. The reason for this discrepancy is unclear, but the highly atherogenic diet used in the study by Menu et al., in comparison with other studies, may have influenced the immune status and inflammatory responses.

## The regulatory mechanisms of NLRP3 inflammasomes

The regulatory mechanisms of NLRP3 inflammasomes may be a potential target for multiple inflammatory diseases including atherosclerosis. The possible mechanisms are the following: (1) modification of the inflammasome components including phosphorylation and ubiquitination, (2) modulation of the NLRP3 inflammasome assembly, (3) the upstream pathways of NLRP3 inflammasome activation, and (4) the destruction of NLRP3 inflammasome complexes. Among NLRP3 inflammasome components, the modification of NLRP3 is critical for inflammasome assembly. The priming signal provided by TLR or IL-1R promotes deubiquitination of NLRP3, which licenses the assembly of NLRP3 inflammasomes [41]. To date, BRCC3 has emerged as the only enzyme that can deubuiquitinate NLRP3 [22], while several E3 ubiquitine ligases such as FBXL12, MARCH7, and TRIM31 are reported to ubiquitinate NLRP3 [42–44]. Further reports suggest that protein kinase A directly inhibits NLRP3 via phosphorylation at Ser 291 and promotes the subsequent ubiquitination [45].

Since oligomerization of NLRP3 inflammasome components is a critical step for inflammasome activation, proteins which interact with the components can modify the assembly of NLRP3 inflammasomes. As a positive regulator of NLRP3 inflammasomes, NEK7 was found to participate in NLRP3 inflammasome complexes [46–48]. Additionally, negative regulatory proteins that prevent assembly of the NLRP3 inflammasomes by direct interaction with components are also reported. For example, CARD-only proteins (COPs) and PYD-only proteins (POPs) are typical molecules that modify NLRP3 inflammasome assembly [49]. These proteins are homologues of caspase-1 and ASC and consist of only CARD or PYD. Among these proteins, POP1 inhibits NLRP3 inflammasome activation by the prevention of ASC oligomerization [50]. Although all the COPs and POPs were first reported as negative regulators of caspase-1 activation [51–53], the roles of COPs and POPs in NLRP3 inflammasome activation remain controversial. In this regard, we previously found that CARD16 induces caspase-1 activation by promoting filamentous assembly of caspase-1 [54]. Furthermore, β-arrestin-2, which functions as a downstream scaffold protein of G protein-coupled receptor 120 (GPR120), is suggested to be a potential negative regulator of NLRP3 inflammasome assembly. Yan et al. reported that the activation of GPR120 by polyunsaturated fatty acids inhibits NLRP3 inflammasomes via the direct interaction between β-arrestin-2 and NLRP3 [55].

The upstream pathways for NLRP3 inflammasome activation are also potential targets. Since mitochondria-derived ROS plays essential roles in the activation of NLRP3 inflammasomes, clearance of damaged mitochondria by autophagy (mitophagy) can inhibit the activation of NLRP3 inflammasomes [56]. Indeed, accelerated atherosclerotic lesion with enhanced NLRP3 inflammasome activation was observed in autophagy-defective Atg5-deficient mice [57]. Furthermore, lysosome biogenesis may protect against crystal-induced NLRP3 inflammasome activation. Activation of lysosome biogenesis by overexpression of TFEB, which regulates lysosome and autophagy-related genes, inhibits NLRP3 inflammasome activation induced by cholesterol crystals and attenuates the progression of atherosclerosis [58]. However, the regulatory mechanism for the destruction of NLRP3 inflammasomes is largely unknown. Although it is suggested that autophagy promotes destruction of the NLRP3 inflammasome complex in a ubiquitin-dependent manner [59], the regulatory mechanism to ubiquitinate the inflammasome complex is undetermined. Thus, the regulatory mechanisms of the NLRP3 inflammasomes are not fully elucidated. Further studies are necessary for the NLRP3 inflammasomes to be therapeutic targets in inflammatory diseases.

## Conclusion

Accumulating evidence suggests that NLRP3 inflammasomes play an essential role in the progression of inflammatory diseases. Lysosome-destabilizing particles, including crystals and particulate matter, are common molecules that trigger activation of the NLRP3 inflammasomes in various diseases. In the progression of atherosclerosis, cholesterol crystals or calcium phosphate crystals are involved in NLRP3 inflammasome-mediated inflammatory responses. Further study clarifying the molecular mechanisms of NLRP3 inflammasome activation would serve to develop a novel therapeutic approach to atherosclerosis and other inflammatory diseases, which are caused by crystals and particulate matter.

## Abbreviation

AIM2: Absence in melanoma 2
ASC: Apoptosis-associated speck-like protein containing a caspase recruitment domain
CAPS: Cryopyrin-associated periodic syndrome
CARD: Caspase recruitment domain
CINCA: Chronic infantile neurological cutaneous and articular syndrome
CLR: C-type lectin receptors
COPs: CARD-only proteins
CPPD: Calcium pyrophosphate dehydrate
DAMPs: Damage/danger-associated molecular patterns
FCAS: Familial cold autoinflammatory syndrome
GPR: G protein-coupled receptor
GSDMD: Gasdermin D
IFI16: IFN-g-inducible protein 16
IL: Interleukin
LDL: Low-density lipoprotein
LRR: Leucine-rich repeats
MSU: Monosodium urate
MWS: Muckle–Wells syndrome
NLR: Nucleotide-binding oligomerization domain-like receptor
NLRP3: NLR family, pyrin domain containing 3
PAMPs: Pathogen-associated molecular patterns
POPs: PYD-only proteins
PRR: Pattern recognition receptor
PYD: Pyrin domain
RLR: Retinoic acid-inducible gene-I-like receptors
ROS: Reactive oxygen species
TLR: Toll-like receptors
